# Diverticular Disease and Rifaximin: An Evidence-Based Review

**DOI:** 10.3390/antibiotics12030443

**Published:** 2023-02-23

**Authors:** Anna Piccin, Marco Gulotta, Stefano di Bella, Paola Martingano, Lory Saveria Crocè, Mauro Giuffrè

**Affiliations:** 1 Department of Medical, Surgical and Health Sciences, University of Trieste, 34149 Trieste, Italy; 2 Infectious Disease Department, Azienda Sanitaria Universitaria Giuliano-Isontina (ASUGI), 34128 Trieste, Italy; 3 Department of Radiology, Azienda Sanitaria Universitaria Giuliano-Isontina (ASUGI), 34128 Trieste, Italy; 4 Liver Clinic, Azienda Sanitaria Universitaria Giuliano-Isontina (ASUGI), 34128 Trieste, Italy; 5 Department of Internal Medicine, Yale School of Medicine, Yale University, New Haven, CT 06510, USA

**Keywords:** diverticulosis, diverticular disease, acute diverticulitis, rifaximin, fiber supplementation, microbiota, diverticular bleeding, diverticulitis prevention, diverticulitis prophylaxis

## Abstract

There have been considerable advances in the treatment of diverticular disease in recent years. Antibiotics are frequently used to treat symptoms and prevent complications. Rifaximin, a non-absorbable antibiotic, is a common therapeutic choice for symptomatic diverticular disease in various countries, including Italy. Because of its low systemic absorption and high concentration in stools, it is an excellent medicine for targeting the gastrointestinal tract, where it has a beneficial effect in addition to its antibacterial properties. Current evidence shows that cyclical rifaximin usage in conjunction with a high-fiber diet is safe and effective for treating symptomatic uncomplicated diverticular disease, while the cost-effectiveness of long-term treatment is unknown. The use of rifaximin to prevent recurrent diverticulitis is promising, but further studies are needed to confirm its therapeutic benefit. Unfortunately, there is no available evidence on the efficacy of rifaximin treatment for acute uncomplicated diverticulitis.

## 1. Background

Diverticula are small, pouch-like protrusions that can occur in the wall of the human gastrointestinal tract, with higher prevalence in the large intestine, given the fact that they represent the most frequent anatomical alteration in the human colon [1]. The process that leads to the development of diverticula is not completely elucidated but is presumably related to age-related changes in the architecture of the colonic wall, which include reduced elasticity and deposition of immature collagen fibers in the extracellular matrix [2]. The colonic wall involves the mucosa, submucosa, muscular, and outer serosal layers. In Western populations, the outpouchings do not involve the muscular layer of the colonic wall, but only the mucosal and submucosal layers, and, therefore, they are defined as “false” or “pseudodiverticula” [3]. However, in Eastern populations, the outpouchings involve all the layers of the colonic wall, and are, therefore, referred to as “true” diverticula [3].

**Figure 1:**
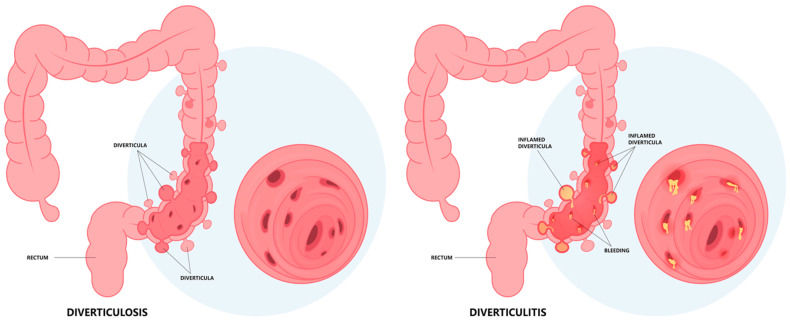
Diverticular disease is characterized by the presence of diverticula, which are small outpouchings in the lining of the colon. In most cases, diverticulosis is asymptomatic, but some people may experience changes in bowel habits such as constipation or diarrhea and vague abdominal symptoms that in same rare cases can resemble acute appendicitis, in a condition referred to as acute diverticulitis.

Diverticular disease (DD) is a term generally used to encompass several clinical scenarios, ranging from diverticulosis to acute diverticulitis (Figure 1). In relation to potential clinical scenarios, most subjects with colonic diverticulosis remain asymptomatic (diverticulosis), while approximately 20% experience abdominal symptoms and possible complications, including episodes of diverticulitis or diverticular bleeding [3].

The symptoms of diverticular disease can be varied, ranging from chronic, recurring gastrointestinal symptoms such as those of irritable bowel syndrome (e.g., abdominal pain and/or discomfort, alteration of bowel movements, and bloating) that characterize symptomatic uncomplicated diverticular disease (SUDD) to acute symptoms/signs analogous to appendicitis (e.g., fever, acute abdominal pain, and leukocytosis) that characterize acute diverticulitis [4]. SUDD is defined as the concomitant presence of diverticula and symptoms of diverticular disease, in the absence of macroscopic signs of colonic inflammation [4]. In approximately 4% of cases, SUDD can progress to acute diverticulitis, characterized by inflammation of one or more diverticula, and can present in its uncomplicated or complicated form (with the presence of abscesses, perforation, fistulas, stenosis, or peritonitis); approximately one third of affected patients may experience recurrent episodes of diverticulitis [1]. Diverticular hemorrhage is the result of the rupture of diverticula-associated arteries, leading to colonic bleeding, and involves less than 5% of patients with diverticulosis [1]. 

### 1.1. Epidemiology and Risk Factors

The exact frequency of occurrence of diverticulosis and diverticular disease remains unknown, particularly because diverticulosis can often be present without symptoms and only discovered incidentally. Age, geographic location, and lifestyle appear to be the most crucial determinants of diverticulosis prevalence. 

The prevalence of diverticulosis is <5% in individuals younger than 40 years of age and can increase to 50% among individuals older than 65 years of age [2]. As per the data obtained from the United States in the year 2009, it was found that the prevalence of diverticulosis among individuals aged 50–59 years was 32.6% and 71.4% among those aged 80 years and above [5]. Additionally, it was observed that in the United States and Europe, diverticulosis was most frequently detected in the left and sigmoid colon (90%), while in Asia, it was more commonly found in the ascending or right colon (75–85%) [6,7,8]. It is estimated that approximately 25% of individuals with diverticulosis will develop SUDD, and that a small proportion of these patients will develop acute diverticulitis (<1% over a 7–11-year period of observation), 20% of patients with acute diverticulitis will experience recurrent episodes, and 12% of patients presenting with diverticulitis will have a complication (such as perforation, abscess, or fistula), resulting in an overall 30-day mortality rate of 8.7% [6,9,10,11].

The differences in diverticulosis prevalence may be related to the low-fiber diet consumed in Western countries, which has been pathogenically linked to increased intraluminal pressure that promotes diverticula formation. [2] A Japanese study highlighted the potential risk of gender, age, tobacco and alcohol consumption, weight gain, and high triglyceride levels [7]. The importance of diet in the development of diverticulitis has been shown. Studies showed that consuming high-fiber foods, eating nuts (>2 times per week) and popcorn (<2 times per week), and a vegetarian diet reduced the risk of diverticulitis, whereas consumption of red meat increased the risk of diverticulitis [1,12,13]. In terms of lifestyle, physical activity reduced the risk of diverticulitis, while BMI ≥ 30 kg/m^2^ and smoking ≥ 15 cigarettes per day increased the risk of diverticulitis [1,12,13]. Medications have also been found to increase the risk of diverticulitis, such as the intake of non-steroidal anti-inflammatory drugs (NSAIDS) ≥ 2 times per week, corticosteroids, opiate analgesics, and postmenopausal hormones [1,12,13,14]. 

### 1.2. Pathogenesis

Several hypotheses have been proposed to explain the pathogenesis of diverticulosis and the progression to diverticular disease. Diverticulosis is hypothesized to be the result of neuromuscular abnormalities such as alteration in connective tissue and collagen metabolism (e.g., altered elastin and increased tissue metalloproteinases) and the enteric nervous system (e.g., reduced number of glial cells and intestinal pacemaker cells), in the setting of increased intraluminal pressure. Alteration in neuromuscular motility also affects colonic motility, by determining non-propulsive spastic contractions which cause “closed chambers of segmentation” where the intraluminal pressure increases according to Laplace’s law [1]. The consequence is represented by herniation of the colonic mucosa in points of greater weakness of the colonic wall [15,16]. Nevertheless, additional changes or precipitating factors might be necessary for the development of symptoms or complications. 

SUDD can result from changes in the gut microbiota caused by food, resulting in persistent, low-grade inflammation mediated by tachykinins. Dietary fiber, for example, boosts intestinal microbiota diversity through bacterial synthesis of short-chain fatty acids (SCFAs) [17,18,19], which improves mucosal barrier and local immunological function. Two cross-sectional studies (diverticulitis group vs. healthy control group) found a decreased level of SCFA-producing Clostridia, and increased levels of strains associated with pro-inflammatory effects, such as *Marvinbryantia* spp. and *Subdoligranulum* spp. [20,21]. In addition, the feces of patients with diverticular disease showed lower levels of anti-inflammatory strains of bacteria such as *Fusobacterium* and *Lactobacillaceae* [22]. However, changes in microbiota diversity do not play a role in diverticula formation. In fact, Van Rossen et al. [23] did not find significant differences in microbiota between patients with asymptomatic diverticulosis and those without diverticula.

In addition, fecal stasis and fecal impaction (trapping of feces in the diverticular sac) can promote fecalith (i.e., hardstone mass of feces) formation that can obstruct the diverticulum leading to bacterial stasis and local trauma, followed by inflammation/ischemia and, eventually, diverticular perforation [15]. 

### 1.3. Diagnosis: Clinical Findings, Laboratory, Imaging, and Endoscopy

Diverticulosis is generally discovered by chance during a colonoscopy or an abdominal radiological examination. At the same time, diverticular disease must be diagnosed using a combination of clinical, radiographic and laboratory data.

Typical clinical findings include intermittent abdominal pain in the lower left quadrant associated with constipation, diarrhea or occasionally abundant rectal bleeding and, on physical examination, tenderness in the lower left quadrant (in a small percentage of patients and in people of Asian origin, pain and tenderness may be localized in the lower right quadrant) [4]. However, there is a poor correlation between clinical findings and the severity of the disease [11]. 

Biomarkers can be used to establish a clinical diagnosis as well as to assess the severity of a disease and track its course. Because of the importance of inflammation in the pathogenesis of diverticular disease, pro-inflammatory indicators such as C-reactive protein (CRP), erythrocyte sedimentation rate, leukocyte count, fecal calprotectin, and procalcitonin may be possible biomarkers [1]. Based on the available data, CRP appears to be the most useful biomarker for diverticulitis. Kechagias et al. [24] found that high CRP values were the best predictor of severe complications in patients with acute diverticulitis. Furthermore, blood CRP values have been linked to the clinical and histological severity of diverticulitis [24]. CRP levels less than 50 mg/L may suggest acute uncomplicated diverticulitis, however CRP levels greater than 200 mg/L may signal complications such as perforation with peritonitis or abscesses [24]. Two other studies confirmed these findings, stating that CRP levels are directly related to the severity of the disease and recurrence rates, especially for CRP higher than 150–200 mg/L [25,26]. However, CRP should be used with caution as a predictor of inflammation severity if there are concomitant conditions that may affect its baseline levels, such as cardiovascular disease, diabetes, and obesity [27,28]. 

**Figure 2:**
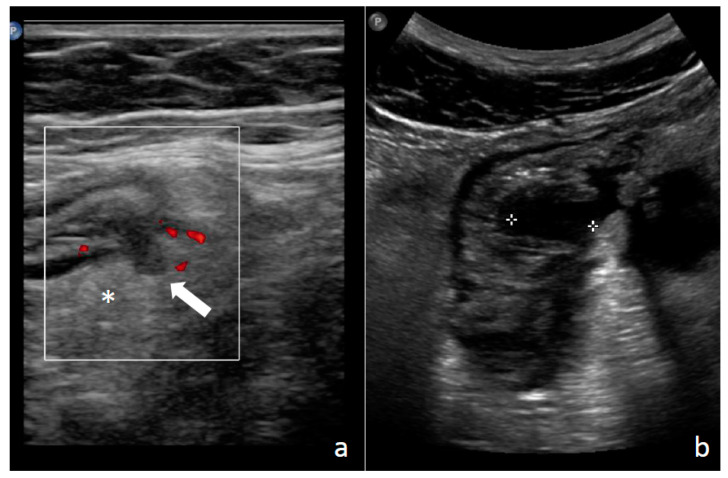
Diverticulitis on ultrasound imaging: (**a**) in the site of tenderness descending colon show a diverticulum as a bowel outpouching (arrow) surrounded by echogenic and non-compressible fat (*) with mild hypervascularization on power Doppler study; (**b**) a localized abscess appears as a hypoechoic fluid collection (between calipers) inside the thickened colonic wall.

However, imaging, such as ultrasonography, computed tomography (CT), or magnetic resonance imaging (MRI), is crucial in the diagnosis of acute diverticulitis. For instance, typical ultrasonography findings in acute diverticulitis include an hypoechogenic thickening of the bowel wall and surrounding inflammation that appears as a hyperechogenic rim, fluid collection, or localized abscesses (Figure 2) [29,30,31,32]. 

**Figure 3:**
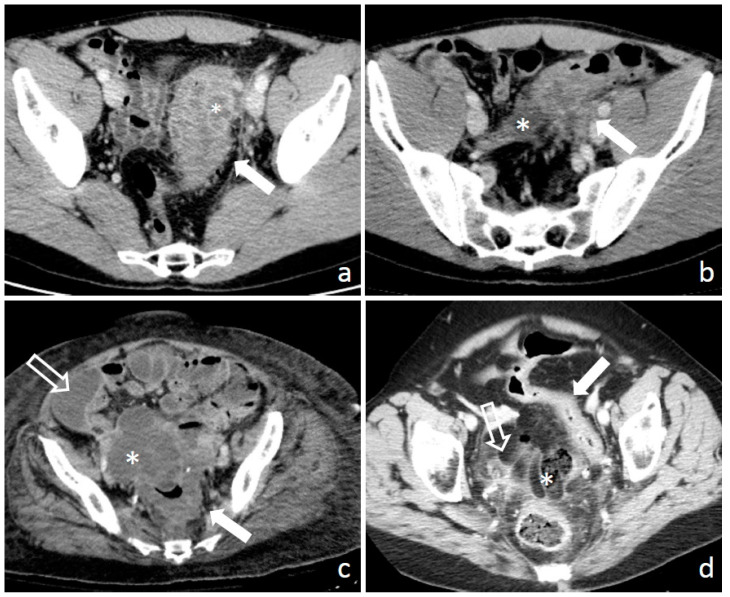
Modified Hinchey classification based on CT imaging: (**a**) Hinchey I—colonic wall thickening (arrow) with pericolic fat stranding and a localized abscess (*); (**b**) Hinchey II—colonic wall thickening (arrow), important fat stranding and a pelvic abscess (*); (**c**) Hinchey III—colonic wall thickening (arrow), with a pelvic abscess (*) and the presence of pus in the abdominal cavity (empty arrow) circumscribed by peritoneal thickening signs of purulent peritonitis; (**d**) Hinchey IV—colonic wall thickening (arrow) with intestinal perforation allowing feces into abdominal cavity (*) associated to fat and peritoneal stranding (empty arrow) signs of fecal peritonitis.

In addition, a CT scan can detect several signs such as distant abscesses, fat stranding, and peritoneal thickening [33]. In 1978, Hinchey et al. [34] published their classification for acute diverticulitis. Hinchey’s classification is the most known and used classification, based on intra-operatory findings, and on the presence of abscesses or peritonitis. The information provided by CT scans led to modifications of the original Hinchey classification [35,36,37,38,39] in four grades: Hinchey I—localized abscess (peri-colonic); Hinchey II—pelvic, distant intra-abdominal or retroperitoneal abscess; Hinchey III—generalized purulent peritonitis (the presence of pus in the abdominal cavity); Hinchey IV—generalized fecal peritonitis (intestinal perforation allowing feces into abdominal cavity) as shown in Figure 3.

Colonoscopy is not routinely indicated in individuals with acute diverticulitis due to the higher risk of bowel perforation, but it should be performed after 6–8 weeks to rule out colorectal cancer [40]. The Diverticular Inflammation and Complication Assessment (DICA) was the first endoscopic classification created to objectively assess the presence of diverticulosis in the colon as well as symptoms of present or previous diverticular inflammation. The DICA classification takes into account four major factors: the location of the diverticula, the number of diverticula in each colonic segment, symptoms of inflammation (edema, hyperemia, erosions, or segmental colitis associated with diverticula SCAD), and complications (i.e., colon rigidity, stenosis, pus, or bleeding) [41].

### 1.4. Principles of Management Strategies in Diverticular Disease

The treatment approach for diverticular disease is customized based on the severity of the disease. The presence of diverticular alone is not a reason to start taking medication, as most people with diverticulosis will not experience symptoms of the disease.

For patients with SUDD, the goal of pharmacological therapy should be to reduce both the intensity and frequency of symptoms and to prevent complications [42,43]. While patients with SUDD may experience mild to moderate pain, bloating, and bowel changes, their quality of life can be significantly impacted. Medical treatment can help improve their quality of life [44]. Treatments for SUDD usually include fiber, antibiotics (such as rifaximin), anti-inflammatory drugs (such as mesalazine or balsalazide), and probiotics, either alone or in combination [42]. Mesalazine (5-aminosalicylic acid) is an established anti-inflammatory drug that has multiple pharmacological effects, though its exact mechanism of action is not fully understood [45]. In diverticular disease, mesalazine may reduce inflammation or modulate pain perception [45]. One double-blind research in individuals with SUDD demonstrated that mesalazine was beneficial at reducing pain during symptomatic flares [46], while another study suggested that it was more effective than a placebo at sustaining remission [47]. A comprehensive evaluation discovered that mesalazine was more beneficial than other frequently used therapies in SUDD [48].

In terms of primary prophylaxis of acute diverticulitis, the available studies on medical treatments are of low quality, and management is often based on experience rather than evidence.

The severity of the illness (e.g., uncomplicated diverticulitis vs. diverticulitis complicated with abscess, perforation, or peritonitis) as well as the co-existence of comorbidities determine how acute diverticulitis is managed. Imaging (such as ultrasonography or CT scan) and laboratory indicators (such as leukocyte count, erythrocyte sedimentation rate, and CRP, which might reflect the severity of the disease) should be used to confirm the clinical suspicion of acute diverticulitis [49,50,51,52]. Normal white blood cell count (WBC) and low CRP (in combination with no fever) may indicate low-risk status in patients with acute uncomplicated diverticulitis (with or without immunocompetence, comorbid disease, and outpatient support), in which case outpatient treatment is feasible and antimicrobial therapy is safe [53,54,55]. Comorbidities, immunosuppression, and the availability of outpatient assistance should all be considered when assessing risk. Outpatients should be provided with a clear liquid diet low in fiber, and antimicrobials should be administered to just a subset of patients [56,57]. Intravenous fluids and antimicrobials should be delivered intravenously to individuals who require hospitalization. Both low-risk and high-risk patients should have symptoms improving within 2–3 days, at which time they should resume a normal diet. If the patient’s condition improves, he or she may be discharged to complete a 7–10-day course of antibiotics at home if necessary [58]. If conservative medical therapy fails, other diagnosis, surgical consultation, and a search for complications may be required.

A thorough evaluation of the severity of the presentation, the co-existence of complications (such as peritonitis or abscesses), and any associated disorders is required for the therapy of acute severe diverticulitis. CT scans are often used to diagnose the complications of diverticular disease using the Hinchey classification. In cases where a patient has a small abscess, treatment typically involves the use of antimicrobial medications and a liquid diet [59]. If this type of antimicrobial therapy is not effective, larger abscesses may need to be treated through percutaneous drainage, which can make it possible to perform elective surgery at a later date [60,61]. In addition to medical treatment, patients with diffuse peritonitis typically require resection surgery. The most appropriate surgical approach will depend on the specific circumstances of the individual case, including factors such as the extent of inflammation in the area where the anastomosis is planned, the patient’s stability and overall health, and any coexisting conditions [62].

## 2. Rifaximin

The use of poorly absorbed antibiotics, which have a high level of availability within the intestinal lumen, is based on the idea that diverticula can facilitate the entrapment of feces in certain individuals, leading to bacterial overgrowth and potential damage to the epithelial lining. This can result in bacterial translocation, mucosal inflammation, and complications that can all contribute to the development of complicated diverticular disease [63]. This idea is supported by studies indicating the existence of intestinal dysbiosis in individuals with SUDD and diverticulitis [22,64,65]. Antimicrobial drugs have also been found to reduce colonic hydrogen production and gas-related symptoms, and they can also increase the mean weight of stools in people with a constant fiber intake, likely due to reduced fiber degradation due to a decrease in the bacterial population [66]. These findings lend support to the use of antibiotics in the treatment of diverticular illness, since decreased gas generation and increased fecal volume can both lower intraluminal pressure and relieve symptoms, as well as reduce the size and number of diverticula [67].

Rifaximin, the most studied and used antibiotic in the setting of diverticulosis and diverticular disease, is a non-aminoglycoside semisynthetic antibiotic derived from the natural antibiotic rifamycin and a structural analog of rifampin [68,69], characterized by the addition of a pyridoimidazole ring, that makes rifaximin a largely water-insoluble, poorly-absorbable antibiotic (blood bioavailability < 0.4% after oral administration) [70], associated with few systemic adverse events and with a safety profile that is comparable to a placebo [71]. Approximately 97% of radiolabeled rifaximin is excreted in the feces as unchanged drug [70] and, according to the findings of Jiang et al. [72], rifaximin concentrations in stool the day after oral administration (800 mg daily for 3 days) were on average 7961 µg/g.

Rifaximin exhibits crystal polymorphism with five forms: α, β, γ, δ, and ε [73]. Laboratory studies have found that these polymorphs have different solubility and dissolution rates. In animal studies, the γ polymorph was found to have the highest systemic bioavailability [73]. In addition to the crystal polymorphs, an amorphous form of rifaximin also exists [73]. Most of the clinical studies have been conducted using rifaximin-α, but the results of these studies may not be directly applicable to generic formulations of rifaximin, which may have higher systemic absorption in both healthy individuals and those with diverticular disease of any severity [74].

### 2.1. Indications, Effects, and Therapeutic Strategies

**Figure 4:**
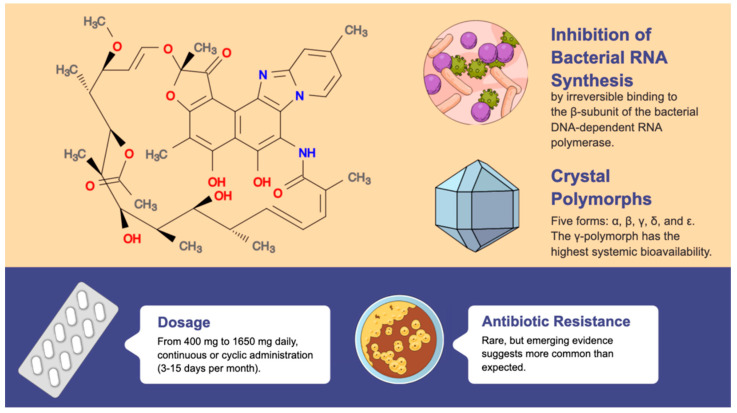
Rifaximin is a non-aminoglycoside semisynthetic antibiotic derived from the natural antibiotic rifamycin. Rifaximin has a largely water-insoluble, poorly absorbable nature, with a blood bioavailability of less than 0.4% after oral administration, with the γ-polymorph having the highest systemic bioavailability. Antibiotic resistance is rare, but more common than expected.

Rifaximin is a medication that is used to treat several different conditions, including small intestine bacterial overgrowth (SIBO), traveler’s diarrhea, and hepatic encephalopathy [75]. Like other rifamycins, rifaximin antimicrobial activity is related to inhibition of bacterial RNA synthesis via irreversible binding to the β-subunit of the bacterial DNA-dependent RNA polymerase, which induces the blockage of the translocation step that normally follows the formation of the first phosphodiester bond, which occurs in the transcription process [76], as shown in Figure 4.

Rifaximin has broad-spectrum activity against Gram-positive and Gram-negative microorganisms [77], including both aerobic and anaerobic bacteria, with a minimum inhibitory concentration MIC for 90% (MIC_90_) of tested pathogens associated with diarrhea ≤ 64 µg/mL (*Campylobacter jejuni*, *Escherichia coli*, *Helicobacter pylori*, *Salmonella* spp., *Shigella* spp., *Yersinia enterocolitica*), [78,79,80,81,82,83,84] that are 80–500 times lower than the actual concentration of rifaximin in feces [72]. It is important to underline that although neither CLSI nor EUCAST breakpoint exist, the potential to reach very high concentrations in stool (around 8000 µg/g after 800 mg daily for 3 days) can in some way reassure clinicians also in the presence of moderately high MIC [72]. Other Gram-negative bacilli (*Klebsiella*, *Proteus*, *Pseudomonas*, *Enterobacter*, and *Acinetobacter species*) that are non-related to diarrhea showed moderately high MIC_90_ (between 4 and 128 µg/mL) [79,85] and this is considered a “plus” from an ecological point of view. Moreover, rifaximin has lower MICs against Gram-positive bacteria, with MIC_90_ < 5 µg/mL, except for methicillin-resistant *Staphylococcus aureus* (MIC_90_ 8–16 µg/mL) and *Enterococcus* (MIC_90_ 4–16 µg/mL) [79,85]. Regarding anaerobes, rifaximin retains a good activity against several anaerobes such as *Bacteroides* spp. (MIC_90_ 0.5–1), *Fusobacterium nucleatum* (MIC_90_ 4), *Veillonella* spp. (MIC_90_ 4), *Clostridium perfringens* (MIC_90_ 0.06), and *Peptostreptococcus* (MIC_90_ 1–16) [82]. It is important to keep in mind that the activity is not satisfactory against *Fusobacterium* other than *nucleatum*, *Prevotella* spp., and *Clostridium*, or *perfringens* [82]. The drug is also usually active against protozoa (*Gardnerella vaginalis*, *Mobilincus* spp., *Cryptosporidium parvum*, and *Blastocystis hominis*) with MIC_90_ ranging from 0.25 to 128 µg/mL [77,83,86,87]. 

Interestingly, rifaximin has a minimal impact on the normal bacteria present in the digestive system. When taken at a dose of 1800 mg per day for 10 days, followed by a 25-day break, and repeated for three cycles, a temporary decrease in certain types of bacteria in the gastrointestinal tract, including *Enterococcus*, *E. coli*, *Lactobacillus* spp., *Bifidobacterium* spp., *Bacteroides* spp., and *C. perfringens*, was found [88,89,90]. However, the levels of these bacteria returned to normal after one month, and there was no increase in the growth of *Candida* species [88,89,90]. Notably, new evidence suggests that rifaximin treatment has a eubiotic effect by promoting the relative abundance of beneficial bacterial strains, such as *Bifidobacteria* and *Lactobacilli* [91].

In addition to its direct antimicrobial activity, rifaximin determines the stabilization of gut epithelial cells and reduction in gut inflammation. In particular, the pre-treatment of epithelial cells with rifaximin determined cellular resistance to infection and bacterial adhesion even after treatment [92]. These effects seem to be related through rifaximin gut-activation of the pregnane X receptor (*PXR*), a nuclear receptor that regulates genes involved in xenobiotic and limited endobiotic deposition and detoxication [93]. 

In terms of therapeutic strategies, there has been significant variability in the dosages and administration methods of rifaximin used in clinical trials for the treatment of diverticular disease including dosages ranging from 400 mg to 1650 mg daily, continuous or cyclic administration (3–15 days per month), and use in combination with other therapies (most commonly fiber). Cyclic administration of rifaximin, in which the drug is taken for a certain number of days each month, is supported by previous research showing that the inhibitory effect of this nonabsorbable antibiotic on fecal microbiota in healthy volunteers is limited to the first two weeks after treatment at a dose of 800 mg per day, and gradually returns thereafter [94,95]. Additionally, a study of patients with ulcerative colitis who took 1800 mg of rifaximin per day in three 10-day treatment periods followed by 25 days of washout found that the concentrations of intestinal microbiota returned to initial values after each washout period, supporting the concept of cyclic administration of this drug [96]. The effectiveness of long-term cyclic administration of rifaximin in SUDD has been studied in several trials lasting 12–24 months [97,98,99,99,99]. However, further research is needed to determine the optimal dosage and duration of treatment cycles for the use of cyclic rifaximin in SUDD.

### 2.2. Long-Term Use and Antimicrobial Resistance

The global spread of antimicrobial resistance is a major public health concern that has been intensified by the overuse and improper use of antibiotics [100]. Antimicrobial stewardship initiatives have been found to enhance patient outcomes, reduce medication adverse events, and reduce antimicrobial resistance worldwide [100]. In the field of digestive diseases, these programs have primarily been implemented in the treatment of cirrhosis patients, who are particularly vulnerable to healthcare-associated infections [101]. Indeed, rifaximin has a low risk of causing systemic resistance due to its poor absorption. Microbial resistance in the gastrointestinal tract is rare and temporary due to the high local concentration of the drug and the lack of horizontal transmission. Furthermore, *Clostridioides difficile* infections are uncommon in individuals receiving rifaximin without predisposing conditions such as hospitalization or immunosuppression, which are uncommon in patients with SUDD [102]. Additionally, the TARGET 3 study discovered that short-term repeat rifaximin therapy had no discernible long-term impact on the microbiological sensitivity of stool to rifaximin, rifampicin, or non-rifamycin antibiotics [102]. 

Reigadas et al. evaluated the incidence of *Clostridioides difficile* infection (CDI) in 388 patients with liver cirrhosis treated with rifaximin. They found CDI in 30.4% of those receiving rifaximin as prophylaxis for hepatic encephalopathy [103]. Resistance to rifaximin was found in 34.1% patients regardless of treatment and in 84.6% of patients receiving rifaximin [103]. Similar concerns were raised for rifampicin-resistant *staphylococci* [104] and *Enterobacterales* [105] after rifaximin treatment.

In conclusion, while rifaximin has a low risk of causing systemic resistance, several studies have highlighted the high prevalence of rifaximin resistant bacteria in patients receiving rifaximin. Therefore, it is important to always consider the risk of developing resistance to rifaximin when prescribing long-term therapy.

### 2.3. Should Rifaximin Be Administered in Diverticulosis?

Colonic diverticulosis itself is not a pathological condition in the absence of progression to diverticulitis. Most patients with diverticulosis will never develop diverticulitis or its complications. However, knowing how to reduce the risk of developing symptoms and/or complications of diverticulitis is important to many patients with diverticulosis. Following an incidental finding of diverticulosis, many patients will ask their doctors for advice on any therapies and how to prevent diverticulitis or its complications. These patients should be informed that the condition is asymptomatic and does not require specific treatment [106]. It should be suggested that patients with diverticulosis must consume a balanced diet including whole foods, fruit, and vegetables; do not need to avoid seeds, nuts, corn, or fruit peels; if they experience constipation and follow a low-fiber diet, they should gradually increase their fiber intake to minimize bloating and abdominal discomfort; increase their fluid intake proportionally to their fiber intake; and if they are constipated, consider using bulk-forming laxatives [106]. It is also suggested that patients who are overweight or obese, do not exercise, or smoke, lose weight, engage in physical activity, and stop smoking to reduce their risk of developing acute diverticulitis and symptomatic diverticulitis [106]. In terms of non-absorbable antibiotics, there are no randomized controlled trial (RCT) or open studies to assess the actual rifaximin efficacy in this setting, with a lack of rationale for drug use in diverticulosis. Of note, in a retrospective study of 248 patients with prior detection of diverticulosis, those treated with rifaximin did not show differences in terms of diverticulitis within six months after treatment [107]. However, between the sixth and twelfth month of treatment, those with rifaximin had significantly lower rates of diverticulitis (4% vs. 32%, *p* < 0.001) [107]. Despite the absence of clear benefit, a recent Italian primary care survey reported that approximately 50% of patients with diverticulosis are treated with rifaximin for primary prophylaxis of diverticular disease [108].

### 2.4. Is Rifaximin Effective at Relieving Symptoms in Individuals with SUDD?

The benefit of rifaximin in SUDD has mainly focused on the reduction of symptoms, particularly in regards to abdominal pain and bloating. A recent retrospective study conducted by the Italian Association for Gastroenterology in Primary Care (GIGA-CP) [109] investigated the cyclical use of Rifaximin 400 mg for 5, 7, or 10 days per month for three months in patients with SUDD. After three months, 47.2% of patients reported pain relief, thus concluding that rifaximin can be used for clinical management of SUDD [109]. In a recent meta-analysis by Bianchi et al. [110] that included four case–control studies for a total of 1660 patients (control group, *n* = 690 vs. treatment group, *n* = 970) treated with rifaximin 400 mg twice a day for seven days each month for 12 months and a dietary fiber supplementation (20 g/die) vs. fiber alone, showed that rifaximin can provide significant symptom relief in a large proportion of patients with uncomplicated diverticular disease compared to control subjects. After 12 months of follow-up, 64.0% of patients treated with rifaximin with a conventional dietary fiber supplement were symptom-free, compared to 34.9% of patients treated with fiber supplementation alone. The increase in total symptom relief after one year was statistically significant and clinically meaningful (29%, NNT: 3). Stallinger et al. [111] reported data on 1003 patients treated with rifaximin for a period of 7–10 days, followed by a 3-week treatment break over a 3-month period. Following the conclusion of the follow-up period, most of the patients (over 90%) reported only moderate or no symptoms, with the exception of 88% who complained of flatulence. After three months of treatment, the treating physician examined the efficacy and tolerability of rifaximin. The efficacy was judged as exceptional in 44% of the cases and very good in 37% of the cases. Tolerability was assessed as outstanding in half of the cases and very good in 34%. During the trial, 24 adverse events were observed in 20 of the 1003 patients, with 6 of these being linked to the administration of rifaximin (0.6% adverse drug response). There were five gastrointestinal adverse effects (flatulence in one patient, stomach discomfort in another, and nausea in three others) and one skin and subcutaneous condition (rash in one patient). This study found that rifaximin had a beneficial effect on overall gastrointestinal symptoms and had a safe profile when used in a cyclic manner. Similar results in terms of symptom relief were reported by other smaller studies [112,113,114,115], with evidence of benefit persistence over a longer period, as demonstrated by a retrospective study that analyzed patients for an 8-year follow-up [113]. As a chronic relapsing disease, SUDD requires cyclical treatment (7–10 days per month) as recommended by various guidelines [106,112,116]. From these data, it emerges that a periodic and scheduled treatment is more effective than an “on demand” one, performed only when symptoms occur, both for symptom control and for preventing long-term complications.

### 2.5. Is rifaximin Useful in Primary Prevention of Diverticulitis in Individuals with SUDD?

Progression from SUDD to acute diverticulitis is not frequent, with recent estimates accounting for 3% of progression rate [117]. In the meta-analysis by Bianchi et al. [110], rifaximin treatment reduced the risk of complications: at 1 year, 1.5% of patients treated with rifaximin plus a standard supplement of dietary fibers experienced complications, compared to 3.2% of patients treated with fiber supplementation alone. However, the 1-year improvement in primary prevention of complications was statistically significant, but not clinically relevant (1.7%, NNT: 59). In addition, cumulative data from placebo-controlled and unblinded trials demonstrated that the incidence of acute diverticulitis was significantly lower in patients who received a combination of rifaximin, and fiber supplementation compared to fiber alone (11/970, 1.1% vs. 20/690, 2.9%; *p* = 0.012). The NNT required to prevent one attack of acute diverticulitis within one year with the rifaximin plus fiber supplementation regimen was 57 [63,97,99,118]. However, in one study, rifaximin did not demonstrate superiority over the placebo in the prevention of acute diverticulitis, with a rate of 2.4% observed in both study groups [118]. Further research is needed to fully understand the effectiveness of rifaximin in preventing diverticulitis in patients with diverticular disease. The available evidence suggests that rifaximin plus fiber may be more effective than fiber alone in preventing acute diverticulitis, although the benefit appears to be small. The high number of individuals needed to treat and the limited number of randomized controlled trials (RCTs) suggest that more research is needed, including larger placebo-controlled trials, to determine the usefulness of this therapeutic regimen in clinical practice.

### 2.6. Is Rifaximin Useful in Secondary Prevention in Patients with Previous Diverticulitis Episodes?

Following an acute episode of diverticulitis, recurrence rates can reach 36% at 5 years. The most implicated risk factors for recurrences seem to be positive family history for diverticular disease and the length of colonic segments involved during the first episode [119]. There is limited research on the use of rifaximin for the secondary prevention (preventing recurrence) of acute diverticulitis. One study found that a combination of rifaximin (400 mg twice a day for 10 days per month) and mesalazine (2.4 g/daily) was more effective than rifaximin alone in improving symptoms and preventing recurrence of diverticulitis in patients in clinical remission [120], while another study found that rifaximin (400 mg twice a day, for seven days a month) plus fiber (3.5 g twice a day) was more effective than fiber alone at preventing recurrence (10.4% vs. 19%, *p* = 0.033) [121]. Tursi et al. [122] found that mesalazine in combination with rifaximin was more effective than rifaximin alone in relieving symptoms and preventing recurrent diverticulitis. This combination treatment involves taking both medications for 7 days each month for a period of 12 months. In one study, the recurrence rate for diverticulitis was 2.7% for the group receiving the combination treatment, compared to 13.0% for the group receiving rifaximin alone [122]. However, the open-label design of these studies, as well as the small number of patients and heterogeneity of the therapeutic regimens used, limit the ability to draw conclusions about the effectiveness of rifaximin for the secondary prevention of acute diverticulitis. *American* guidelines do not consider sufficient evidence to recommend the use of Rifaximin in secondary prevention [123]. The same conclusion was seen in more recent *Italian* guidelines [106]. Additionally, in this case, the problem seems to be related to the heterogeneity of the studies and the low number of patients involved. More randomized controlled studies are needed to determine the usefulness of rifaximin in this setting.

### 2.7. Can Rifaximin Be Used in the Treatment of Uncomplicated Acute Diverticulitis?

Systemic antibiotics are commonly used to manage acute diverticulitis. However, three systematic reviews [124,125,126] have found that systemic antibiotics do not offer clear benefits over supportive care (such as rest and hydration) in uncomplicated acute diverticulitis [126,127,128]. These findings suggest that acute diverticulitis may be more of an inflammatory than an infectious condition. In addition, the use of systemic antibiotics is linked to several downsides, including costs, adverse events, allergic reactions, and antibiotic resistance. A recent observational study compared the effectiveness of systemic antibiotics to observation (no treatment) in patients during the first episode of uncomplicated acute diverticulitis. The study found that recovery time was not significantly different between the two groups, and there were no significant differences in complications, recurrence, surgery, readmission, adverse events, or mortality. However, hospital stays were shorter in the observation group (2 vs. 3 days, *p* = 0.006) [128]. These results suggest that observation without antibiotics may be a viable treatment option for patients with uncomplicated diverticulitis. Rifaximin, a medication with both anti-inflammatory and eubiotic (promoting the growth of beneficial bacteria) properties, may also be a potential treatment option for uncomplicated acute diverticulitis, although more research is needed to confirm its effectiveness.

## 3. Conclusions

Diverticular disease treatment can be difficult, especially when dealing with individuals who have gastrointestinal symptoms and abdominal discomfort. Aside from the main clinical issues discussed in this review, it is crucial to emphasize that the use of medications, including rifaximin, for the primary prevention of diverticulitis in patients with diverticulosis is not supported by therapeutic reasoning and should be avoided. Although the cost-effectiveness of long-term treatment (over two years) has yet to be demonstrated, cyclic use of rifaximin in conjunction with high fiber intake is safe and effective for the treatment of SUDD symptoms, as outlined in Table 1. The use of rifaximin with fiber to prevent diverticulitis recurrence is encouraging, but the minimal therapeutic benefit needs a large and strong randomized controlled study for confirmation. Finally, there is no evidence that rifaximin is effective in the treatment of acute uncomplicated diverticulitis, thus it should not be used for this reason.

## Figures and Tables

**Table 1:** Recommendation on current use of rifaximin in diverticular disease. Adapted from Italian Society of Gastroenterology (SIGE) position paper [106].

Diverticular Disease Severity	Recommendation	Therapeutic Regimen
Diverticulosis	Absence of supporting strong evidence	None
Symptomatic uncomplicated diverticular disease	Yes, in addition to fiber supplementation (e.g., glucomannan 2–4 g/day)	400 mg twice daily for 12–24 months, administered cyclically for 7–10 days each month.
Acute diverticulitis (primary prophylaxis)	Yes, in addition to fiber supplementation (e.g., glucomannan 2–4 g/day)	400 mg twice daily for 12–24 months, administered cyclically for 7–10 days each month.
Acute diverticulitis (secondary prophylaxis)	Yes, absence of supporting strong evidence for fiber supplementation.	400 mg twice daily, administered cyclically for 7–10 days each month.
Uncomplicated acute diverticulitis	Absence of supporting strong evidence	None

## Data Availability

Not applicable.
